# In Vitro Screening of Short Synthetic Antimicrobial Peptides Toward Food-Relevant Microorganisms: Antifungal Activity and Molecular Docking Analysis

**DOI:** 10.3390/microorganisms14092088

**Published:** 2026-09-18

**Authors:** Marselle Marmo do Nascimento Silva, Aline F. Miller Gavin Humphreys, Maria Alice Zarur Coelho, Filipe Smith Buarque

**Affiliations:** 1 Biochemical Engineering Department, School of Chemistry, Federal University of Rio de Janeiro, Rio de Janeiro 21941-853, Brazil; marselle@eq.ufrj.br (M.M.d.N.S.); alice@eq.ufrj.br (M.A.Z.C.); 2 Faculty of Science and Engineering, University of Manchester, Manchester M13 9PL, UK; aline.miller@manchester.ac.uk; 3 CICECO-Aveiro Institute of Materials, Department of Chemistry, University of Aveiro, 3810-193 Aveiro, Portugal

**Keywords:** antimicrobial peptides, food preservation, *Candida albicans*

## Abstract

Food spoilage and foodborne microorganisms remain major challenges for food safety and preservation, stimulating the search for alternative antimicrobial agents compatible with minimally processed and clean-label products. In this study, six short synthetic peptides containing arginine, or both lysine and arginine residues, were evaluated as potential antimicrobial agents against *Staphylococcus aureus*, *Salmonella enterica*, and *Candida albicans*. Minimum inhibitory concentrations were determined by broth microdilution, and molecular docking was performed against *C. albicans* lanosterol 14α-demethylase to provide molecular-level insights. None of the peptides inhibited *S. enterica* within the tested concentration range, indicating limited activity against the Gram-negative bacterium. Peptides 1, 3, 5, and 6 inhibited *S. aureus* only at relatively high concentrations, with MIC values between 1598.7 and 1782.0 µmol L^−1^, without confirmed bactericidal activity. In contrast, peptide 4, FEFKFEFKGGGRGDS, was the only sequence active against *C. albicans*, showing an MIC of 19.93 µmol L^−1^. However, no minimum inhibitory concentration (MIC) was obtained within the tested concentration range, indicating that the observed antifungal effect should be interpreted as inhibitory rather than confirmed fungicidal activity. Docking analysis was consistent with this experimental trend, as peptide 4 exhibited the most favorable predicted binding energy, −8.4 kcal mol^−1^, and established predicted contacts with catalytic site of ERG11, including Lys143, Arg381, His468, Arg469, and Cys470. These findings indicate that antimicrobial activity was strongly sequence- and microorganism-dependent. Peptide 4 represents a promising antifungal lead sequence for further optimization and validation in food-relevant systems.

## 1. Introduction

Food spoilage and foodborne diseases remain major challenges for the food industry, public health systems, and global food supply chains. Microbial contamination can occur at different stages of production, processing, storage, distribution, and consumption, leading not only to economic losses but also to serious risks to consumer health [1,2]. In addition, pathogenic microorganisms may produce toxins or cause infections, underscoring the need for efficient preservation strategies that ensure food safety while maintaining product quality [3]. Conventional food preservation methods, including drying, freezing, thermal processing, irradiation, vacuum packaging, modified-atmosphere packaging, canning, and the addition of chemical preservatives, are widely employed to prevent or delay microbial growth and biochemical deterioration [1]. This strategy may improve microbial control while minimizing negative impacts on the physicochemical and sensory attributes of food products. However, some traditional preservation processes may affect nutritional quality, induce undesirable chemical modifications, or alter consumer perception, especially when synthetic additives are used at high concentrations [4]. Despite this growing interest, the practical application of short synthetic antimicrobial peptides remains constrained by technical challenges, including relatively high inhibitory concentrations for some microorganisms and reduced activity or stability in complex food matrices, reinforcing the need for systematic screening and comparative evaluation of candidate sequences [5].

In recent years, increasing consumer demand for minimally processed foods, clean-label ingredients, and products containing fewer artificial additives has stimulated the search for alternative antimicrobial agents. In this context, bioactive peptides have emerged as promising candidates for food preservation due to their structural versatility, biological activity, and potential compatibility with food matrices [6]. Peptides are short chains of amino acids that may differ widely in sequence, charge, hydrophobicity, amphipathicity, secondary structure, and biological function. Among them, antimicrobial peptides (AMPs) have attracted particular attention because of their ability to inhibit or eliminate bacteria, fungi, and other microorganisms through mechanisms that often differ from those of conventional preservatives and antibiotics [7,8].

AMPs are generally characterized by short amino acid sequences, a net positive charge, and an amphipathic structure that allows selective interaction with microbial envelopes. Their antimicrobial activity is often mediated by electrostatic interactions between cationic residues and negatively charged components of microbial cell surfaces, such as teichoic acids in Gram-positive bacteria, lipopolysaccharides in Gram-negative bacteria, and phospholipids or other anionic constituents of fungal membranes [9,10,11]. After initial adsorption, hydrophobic residues may facilitate peptide insertion into the lipid bilayer, thereby promoting membrane destabilization, increased permeability, pore formation, and cytoplasmic leakage, ultimately leading to cell death [12]. Depending on the peptide sequence and the target microorganism, additional intracellular effects may occur, including interference with nucleic acids, protein synthesis, enzymatic activity, or cell wall biosynthesis [13].

Short synthetic AMPs represent an attractive strategy for food preservation because they can be designed with defined sequences, reproducible composition, and tunable antimicrobial properties. Their relatively small size may facilitate chemical synthesis, sequence optimization, and incorporation into food-related systems, including coatings, packaging materials, edible films, and liquid formulations [14]. Compared with traditional organic acids and other chemical preservatives, antimicrobial peptides may offer specific advantages, such as activity at low concentrations, broad-spectrum activity, and reduced impact on food flavour when properly formulated [15]. Nevertheless, their practical use depends on several factors, including antimicrobial efficacy in complex food matrices, stability under processing and storage conditions, resistance to proteolysis, safety, production costs, and compliance with regulatory requirements [16].

Microorganisms such as *Salmonella enterica*, *Staphylococcus aureus*, and *Candida albicans* are relevant models for evaluating the antimicrobial potential of preservative candidates. *S. enterica* is a Gram-negative foodborne pathogen frequently associated with contaminated meat, poultry, eggs, fresh produce, and processed foods. *S. aureus* is a Gram-positive bacterium of major concern due to its ability to contaminate food products and produce heat-stable enterotoxins. *C. albicans*, although more commonly recognized as an opportunistic fungal pathogen, is also useful as a model yeast-like microorganism for assessing antifungal activity and membrane-targeting effects [17]. The inclusion of microorganisms with distinct cell envelope compositions provides important information on the spectrum of action and possible structure-activity relationships of antimicrobial peptides [18].

The design and selection of short antimicrobial peptides are generally based on physicochemical parameters known to influence biological activity, including cationicity, hydrophobicity, amphipathicity, peptide length, and residue distribution. Arginine- and lysine-containing sequences are often associated with enhanced electrostatic interactions with negatively charged microbial surfaces, while hydrophobic aromatic residues may facilitate membrane partitioning and disruption. Conversely, modifications in sequence arrangement and charge balance may substantially alter peptide selectivity and potency. According to these literature-supported principles, the peptides evaluated in this study were selected as short synthetic sequences with distinct amino acid arrangements to examine the effects of variations in composition and length on antimicrobial behavior.

Despite the growing interest in antimicrobial peptides for food-related applications, several knowledge gaps remain regarding the behavior of short synthetic peptide sequences in microbiologically diverse systems. First, comparatively few studies have evaluated structurally related short arginine- and lysine-containing peptides across microorganisms representing distinct biological barriers, including Gram-positive bacteria, Gram-negative bacteria, and yeast-like fungi. Then, the use of molecular docking to investigate the interaction of food-relevant antimicrobial peptide candidates with fungal targets such as lanosterol 14α-demethylase (ERG11) remains limited. Furthermore, systematic comparison of how peptide length, charge distribution, hydrophobicity, and residue arrangement influence antimicrobial responses across food-relevant microorganisms is still insufficiently explored. Therefore, the present study aimed to comparatively evaluate six previously reported short synthetic peptides against Staphylococcus aureus, *Salmonella enterica*, and *Candida albicans*, to identify sequence-dependent antimicrobial trends and to relate these responses to selected physicochemical properties. In addition, molecular docking with *C. albicans* ERG11 was employed as a complementary computational approach to examine predicted peptide–target interaction patterns associated with the observed antifungal activity.

## 2. Materials and Methods

### 2.1. Peptides and Stock Solution Preparation

Six short synthetic peptides were evaluated in this study: FEFR, FEFEFRFK, FEFKFEFKK, FEFRFEFK, FEFRFEFKK, and FEFKFEFKGGGRGDS. None of these sequences was newly designed for the present investigation [19,20,21,22]. Chromatographic profiles of the peptide sequences evaluated in this study are provided in the Supplementary Materials (Figure S2 in Supporting Information). Their inclusion was based on differences in peptide length, residue arrangement, and the distribution of aromatic, cationic, and acidic amino acids, allowing comparative evaluation of sequence-dependent antimicrobial behavior. No computational peptide design algorithm was used to generate the sequences evaluated in this study.

The peptides were purchased from Biomatik Corporation (Cambridge, ON, Canada) as lyophilized powders. Prior to biological assays, the commercial peptide preparations were subjected to a trifluoroacetic acid (TFA) counterion-exchange treatment following established procedures involving HCl acidification, freezing, and lyophilization. This treatment was applied to reduce the likelihood of residual interference from trifluoroacetate in the antimicrobial assays [23,24,25]. Briefly, each peptide powder was dissolved in filtered distilled water at a 1:1 ratio and homogenized using a vortex mixer. Subsequently, 1 mol L^−1^ HCl was added at a volume 100-fold lower than the water volume, followed by additional homogenization. The peptide solutions were frozen for 1 h and then lyophilized. After this procedure, the resulting powders were used for antimicrobial assays. Stock solutions were prepared by dissolving each peptide in filtered distilled water at a final concentration of 5000 µg mL^−1^. The pH of the peptide solutions was measured after dissolution; however, no pH adjustment was performed to avoid potential changes in peptide structure, ionization state, or biological activity.

### 2.2. Microorganisms and Culture Conditions

The antimicrobial activity of the peptides was evaluated against three microorganisms relevant to food safety and spoilage: the Gram-negative bacterium *Salmonella enterica* subsp. *enterica* serovar Enteritidis ATCC 13076, the Gram-positive bacterium *Staphylococcus aureus* clinical isolate, and the yeast *Candida albicans* clinical isolate. Bacterial colonies were cultivated in Brain Heart Infusion broth, BHI, 53286, Sigma-Aldrich Co., at 35 ± 2 °C for 24 h. *Candida albicans* was cultivated in Sabouraud dextrose broth (S3306, Sigma-Aldrich Co., St. Louis, MO, USA) at 28 ± 2 °C for 48 h. The identity and purity of the microbial cultures were confirmed by Gram staining and microscopic examination of isolated colonies prior to the antimicrobial assays.

### 2.3. Preparation of Bacterial and Yeast Inoculum

Microbial inoculum was prepared according to standardized broth microdilution procedures recommended by CLSI for bacteria and NCCLS for yeasts, with minor adaptations [26]. For bacterial assays, the turbidity of each bacterial suspension was adjusted to an absorbance of 0.08–0.10 at 625 nm using a spectrophotometer (Spectramax M2, San Jose, CA, USA). This turbidity corresponds approximately to 1 × 10^8^ CFU mL^−1^. The standardized suspension was vortexed and diluted 1:20 in Mueller-Hinton broth (70192, Sigma-Aldrich Co.) to obtain the working inoculum. After inoculation into the microplate wells, the final bacterial concentration was approximately 5 × 10^5^ CFU mL^−1^. For *C. albicans*, the turbidity of the yeast suspension was adjusted by spectrophotometric reading at 530 nm. The standardized yeast suspension was vortexed, diluted 1:100 in Sabouraud dextrose broth, and subsequently diluted 1:20 in RPMI-MOPS medium. After inoculation into the wells, the final yeast concentration ranged from 0.5 × 10^3^ to 2.5 × 10^3^ CFU mL^−1^.

### 2.4. Minimum Inhibitory Concentration Assay

**Figure 1:**
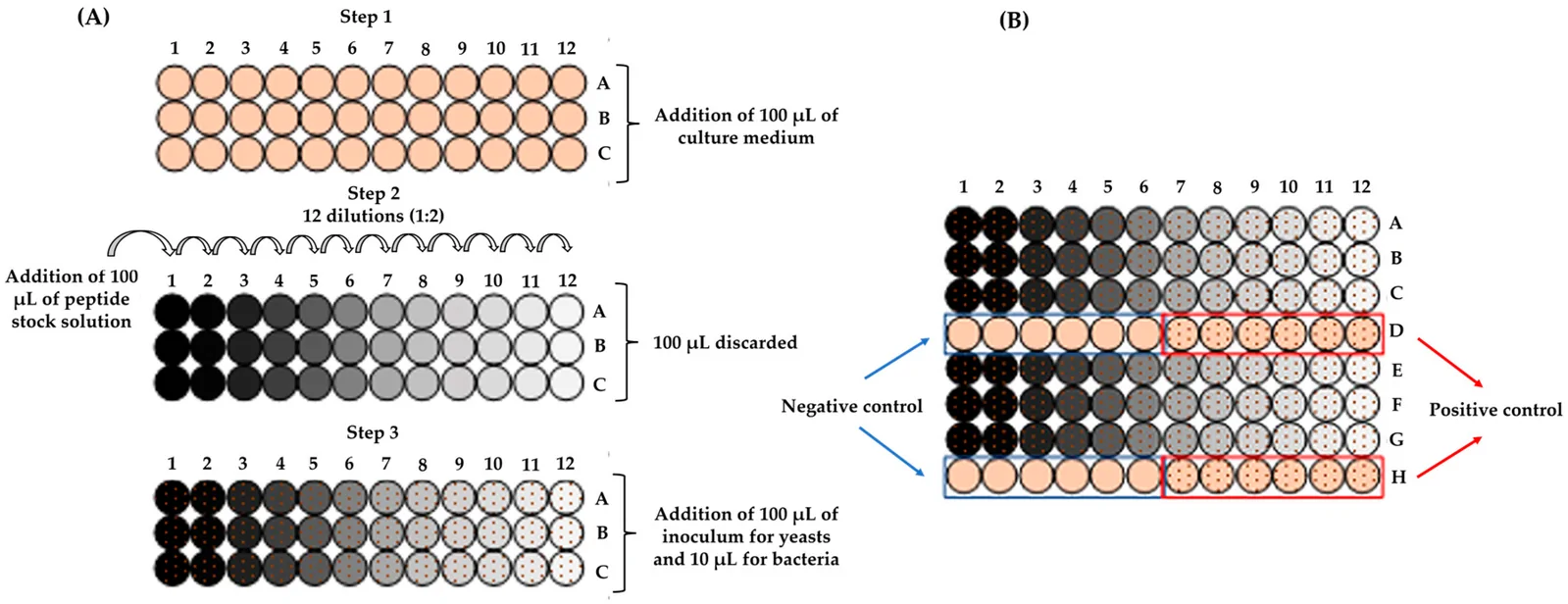
Protocol for determination of MIC using a peptide stock solution against bacteria/yeast (**A**); Scheme of a 96-well plate testing two different peptides (one at lines A–D and other at lines E–H), with positive and negative controls (**B**). Lines A–D correspond to the first peptide, whereas rows E–H correspond to the second peptide, with each peptide concentration tested in replicate wells. Positive growth controls consisted of inoculated medium without peptide, whereas negative controls consisted of culture medium without microbial inoculum.

The minimum inhibitory concentration (MIC) of each peptide was determined using the broth microdilution method in 96-well microplates, following international standardized protocols for bacteria and yeasts. For each peptide, serial two-fold dilutions were prepared from a 5000 µg mL^−1^ stock solution in the appropriate culture medium. Mueller-Hinton broth was used for bacterial assays, and RPMI-MOPS medium (pH 7.2) was used for yeast assays. The concentration range tested was limited by peptide solubility in the corresponding assay medium. For bacterial assays, 100 µL of peptide dilution in Mueller-Hinton broth was added to each well, followed by inoculation with 10 µL of bacterial suspension. For yeast assays, peptide dilutions were prepared in RPMI-MOPS medium and inoculated with 100 µL of fungal suspension. Microplates containing bacteria were incubated at 37 °C for 24 h, whereas yeast microplates were incubated at 28 °C for 48 h (Figure 1A). Pure culture medium was used as the negative control, and the inoculated medium without peptide was used as the positive growth control. After incubation, 15 µL of PrestoBlue^®^ Cell Viability Reagent (A13261, Thermo Fisher Scientific, Waltham, MA, USA) was aseptically added to each well, followed by incubation for 10 min at 37 °C for bacteria or 28 °C for yeast. MIC endpoints were determined visually based on the reduction-dependent color response of PrestoBlue (Figure 1B). The sterility control, containing culture medium without microbial inoculum, was required to retain the blue no-growth appearance, whereas the peptide-free inoculated growth control was required to develop a pink color indicative of microbial growth. Peptide-containing wells retaining a blue or predominantly blue/purple appearance comparable to the sterility control were classified as showing no detectable growth, whereas wells developing a pink coloration comparable to the growth control were classified as growth-positive. The MIC was defined as the lowest peptide concentration showing the no-growth endpoint consistently across replicate wells. Representative endpoint photographs are provided in Figure S1 of the Supplementary Materials.

### 2.5. Minimum Bactericidal and Fungicidal Concentration Assays

Minimum bactericidal concentration (MBC) and minimum fungicidal concentration (MFC) were determined from wells showing no visible microbial growth in the MIC assay. Approximately 3 µL from each clear well, corresponding to the MIC and higher peptide concentrations, was transferred onto agar plates. For bacterial assays, aliquots were plated onto BHI agar and incubated at 37 °C for 24 h. For C. albicans, aliquots were plated onto Sabouraud dextrose agar and incubated at 28 °C for 48 h. MBC or MFC was defined as the lowest peptide concentration that prevented visible colony formation after incubation, corresponding to the concentration required to eliminate the target microorganism under the experimental conditions used.

### 2.6. Molecular Docking

Molecular docking simulations were performed to compare the predicted interaction profiles of the six synthetic peptides with lanosterol 14α-demethylase from *Candida albicans*, encoded by the *ERG11* gene. *ERG11* was selected because it is a key enzyme in the ergosterol biosynthetic pathway and represents a well-established molecular target in antifungal research. The docking analysis was restricted to a fungal receptor because peptide 4 was the only sequence showing relevant activity against *C. albicans* under the experimental conditions evaluated [27]. The three-dimensional structure of *C. albicans* lanosterol 14α-demethylase was retrieved from the AlphaFold Protein Structure Database using the UniProt accession number P10613. The AlphaFold model of *C. albicans* ERG11 (UniProt P10613) presented a global pLDDT score of 95.21, indicating very high overall structural confidence. Model quality was additionally considered using the AlphaFold pLDDT and predicted aligned error (PAE) confidence metrics, which provide information on local residue-level confidence and the relative positioning of protein regions, respectively. The structural model was visually inspected before docking, with particular attention to the predicted quality of the receptor region explored in the calculations. The three-dimensional structures of peptides 1–6 were generated from their respective amino acid sequences using AlphaFold/ColabFold. For each peptide, the highest-ranked model was selected according to the confidence metrics provided by the prediction platform. The selected structures were downloaded in CIF format and subsequently converted to PDB format [28]. As the receptor model was obtained directly from AlphaFold, no explicit heme cofactor was present in the structural model used for docking, and no heme group was subsequently introduced during receptor preparation. Therefore, predicted contacts involving residues surrounding the heme-binding region were interpreted as peptide–protein interactions within the ERG11 functional environment rather than as direct interactions with the heme cofactor.

Docking calculations were performed using the AutoDock Vina 1.1.2 program. Before to docking, the *ERG11* structure was prepared using AutoDockTools following the standard receptor-preparation workflow. Water molecules were removed, hydrogens and atomic charges were assigned during preparation, and the receptor was converted from PDB to PDBQT format for AutoDock Vina calculations. A blind-docking strategy was adopted, with a search space covering the receptor structure, because no experimentally validated peptide-binding site has been established for the evaluated sequences. The grid box was centered at x = 1.038, y = 3.324, and z = −1.401 Å, with dimensions of 126 × 126 × 126 Å. The exhaustiveness parameter was set to 100 to increase conformational sampling during the global search and to provide a more extensive exploration of possible peptide-binding regions. Ten poses were generated for each peptide using identical docking parameters. No predefined docking-score cutoff was applied to classify poses as valid or invalid; the generated poses were ranked according to the AutoDock Vina scoring function, and the highest-ranked pose, corresponding to the lowest Vina score for each peptide, was selected for comparative interaction analysis [29].

## 3. Results and Discussion

### 3.1. Antimicrobial Activity of Short Synthetic Peptides Against Food-Relevant Microorganisms

The antimicrobial potential of six short synthetic peptides was evaluated against three microorganisms relevant to food safety and spoilage: the Gram-positive bacterium *Staphylococcus aureus*, the Gram-negative bacterium *Salmonella enterica*, and the yeast *Candida albicans*. These microorganisms were selected for their distinct cell structures and surface compositions, enabling the evaluation of the peptides’ activity against different biological barriers. The minimum inhibitory concentration (MIC) values obtained by broth microdilution are summarized in Table 1. The tested concentration range was limited by peptide solubility in the culture medium, varying from 2272.7 to 1.11 µg mL^−1^ for bacteria and from 1250 to 0.61 µg mL^−1^ for yeast.

The antimicrobial response was strongly dependent on both peptide sequence and target microorganism. None of the peptides inhibited the growth of *S. enterica* within the tested concentration range, whereas partial inhibitory activity was observed against *S. aureus* for peptides 1, 3, 5, and 6 [30]. A remarkably different profile was observed for *C. albicans*, for which only peptide 4, FEFKFEFKGGGRGDS, displayed relevant antifungal activity, with an MIC of 19.93 µmol L^−1^. These results demonstrate a restricted and microorganism-dependent antimicrobial profile, rather than broad-spectrum activity, under the conditions evaluated [31]. For *S. aureus*, peptides 1, 3, 5, and 6 inhibited growth only at relatively high concentrations, with MIC values of 1782.0, 1630.8, 1782.0, and 1598.7 µmol L^−1^, respectively. Peptides 2 and 4 did not inhibit *S. aureus* within the evaluated range, presenting MIC values higher than 3487.3 and 1159.4 µmol L^−1^, respectively. Although these results demonstrate that some sequences were able to interfere with the growth of this Gram-positive bacterium, the concentrations required were considerably higher than those commonly reported for highly optimized antimicrobial peptides [32]. For example, Festa et al. [33] reported that the peptide 1018-K6, a 12-residue cationic peptide evaluated against foodborne pathogens, has been reported to exhibit low micromolar/high-potency antibacterial activity, with MIC values against *Salmonella enterica* strains ranging from 8 to 64 µg mL^−1^ and MBC values of 16 to 128 µg mL^−1^.

The lower antibacterial activity observed for the peptides evaluated here can also be understood by comparing their structural features with those of previously reported active antimicrobial peptides. The 1018-K6 peptide is a 12-residue cationic sequence (VRLIVKVRIWRR-NH_2_) containing multiple lysine and arginine residues and a substantial proportion of hydrophobic amino acids. This composition favours both electrostatic attraction to negatively charged bacterial surfaces and subsequent membrane association. In addition, 1018-K6 has been reported to adopt an α-helical conformation in membrane-mimetic environments, providing a more organized amphipathic arrangement for membrane interaction [33]. A similar contrast is observed for KRR-N1-5W6L (KRRLIVTWLMK), an 11-residue peptide specifically optimized from the buffalo-colostrum-derived peptide N1 by introducing additional positively charged and hydrophobic residues. This modification increased cationicity and hydrophobic character and resulted in markedly enhanced antibacterial activity, with an MIC of 32 µM against *S. enterica*. In contrast, the peptides evaluated in the present study contain recurrent glutamate residues, which introduce negatively charged side chains and reduce the overall cationic character provided by lysine and arginine. Peptides 1, 2, and 5 contain equal numbers of basic and acidic residues, whereas peptides 3 and 6 contain only one additional basic residue relative to acidic residues [34]. Consequently, their charge distribution is substantially less cationic than that of highly active sequences such as 1018-K6 and KRR-N1-5W6L. This reduced cationic density may weaken the initial electrostatic attraction to negatively charged bacterial membranes and thereby limit subsequent membrane association and disruption. Thus, the weaker antibacterial activity observed here is consistent with a less optimized balance between positive charge, hydrophobicity, residue distribution, and amphipathic organization rather than with peptide length alone [32].

Regarding peptide applications in food matrices, the relatively high MIC values observed against *S. aureus* also pose practical limitations for direct use in food systems. At such concentration levels, economic feasibility may become unfavourable, especially considering peptide synthesis and formulation costs. In addition, peptide performance in foods may be further reduced by interactions with proteins, salts, lipids, and other matrix components, potentially requiring even higher effective concentrations. Under these conditions, sensory impact and formulation compatibility would also need to be carefully assessed. Therefore, the inhibitory response observed against *S. aureus* should be interpreted cautiously and does not, by itself, support immediate applicability in food preservation. The inhibitory profile observed in the present study may be associated with the physicochemical properties of the tested peptides. Classical antimicrobial peptides usually combine a positive net charge, sufficient hydrophobicity, and amphipathic organization, which together favour electrostatic attraction to microbial surfaces followed by membrane insertion or destabilization [35]. In the evaluated sequences, the presence of phenylalanine contributes a hydrophobic character, while lysine and arginine provide cationic sites [10]. However, the recurrent presence of glutamate residues introduces negative charges, potentially reducing the overall cationic density of the molecules. This charge balance may weaken the initial electrostatic interaction with negatively charged microbial membranes, especially when compared with highly cationic AMPs rich in lysine, arginine, and hydrophobic residues [36].

The results obtained for *S. aureus* are consistent with the general observation that Gram-positive bacteria are often more susceptible to cationic antimicrobial peptides than Gram-negative bacteria, mainly due to the absence of an outer membrane. In Gram-positive cells, the cytoplasmic membrane is protected by a thick peptidoglycan layer containing negatively charged teichoic and lipoteichoic acids, which can promote the adsorption of cationic peptides [10,37]. Nevertheless, peptide access to the membrane and subsequent permeabilization still depend on sequence length, charge distribution, hydrophobicity, and conformational flexibility. Therefore, the inhibition of *S. aureus* only at high concentrations suggests that the evaluated peptides may interact with the bacterial envelope but do not possess an optimized balance of physicochemical properties for strong bactericidal activity [32]. Importantly, no minimum bactericidal concentration (MBC) could be determined for *S. aureus* under the experimental conditions used. This indicates that, although some peptides inhibited visible bacterial growth, they could not kill 99.9% of the bacterial population within the tested concentration range. This distinction is relevant since MIC values indicate growth inhibition, but do not necessarily reflect cell death. In practical terms, for application as food preservatives, peptides with only inhibitory activity may still be useful if they delay microbial proliferation; however, bactericidal activity would be desirable for stronger preservation performance, particularly in high-risk food matrices [38].

The absence of inhibitory activity against *S. enterica* was one of the most relevant findings of this study. For all six peptides, MIC values were higher than the maximum concentrations evaluated, indicating that *S. enterica* was resistant to the tested sequences under the assay conditions. This behavior can be explained, at least in part, by the structural complexity of the Gram-negative outer membrane [39]. The outer membrane contains lipopolysaccharides that act as a permeability barrier and can restrict peptide access to the inner cytoplasmic membrane. Although cationic peptides may interact with negatively charged LPS, effective antimicrobial action requires sufficient binding, displacement of divalent cations, outer membrane permeabilization, and subsequent disruption of the inner membrane. The literature highlights that the outer membrane is a major determinant of Gram-negative resistance to many antimicrobial compounds, including cationic antimicrobial peptides [10]. This result also agrees with observations reported for food-grade bacteriocins and antimicrobial peptides used in preservation. Nisin, one of the most established peptide-based food preservatives, is mainly effective against Gram-positive bacteria, whereas its activity against Gram-negative bacteria generally requires combination with treatments that destabilize the outer membrane. Pediocin PA-1 is also reported to be inactive against Gram-negative bacteria, reinforcing that the outer membrane represents a major limitation for peptide-based preservation strategies targeting *Salmonella* and other Gram-negative pathogens. In contrast, other optimized peptides have shown strong activity against *S. enterica*. Festa et al. [33] also evaluated the peptide 1018-K6 against 42 *S. enterica* strains and reported MIC values ranging from 8 to 64 µg mL^−1^, with most strains showing MIC values between 16 and 32 µg mL^−1^. The same study also reported bactericidal and antibiofilm activity, supporting the potential use of optimized AMPs in active packaging and food disinfection systems. More recently, Ma et al. [34] verified that KRR-N1-5W6L, a modified peptide derived from buffalo colostrum whey, was reported to inhibit E. coli O157:H7 and *S. enterica* with an MIC of 32 µM, while also showing thermal and pH stability relevant to food applications.

Among all results, the most promising activity was observed for peptide 4 against *C. albicans*. Peptide 4, FEFKFEFKGGGRGDS, inhibited yeast growth at 19.93 µmol L^−1^, whereas all other peptides showed MIC values above the maximum concentrations tested. This selective antifungal activity is notable since peptide 4 was not active against *S. aureus* or *S. enterica* within the tested range, suggesting that its sequence may interact preferentially with fungal cellular structures rather than bacterial envelopes. The presence of the GGGRGDS segment may influence peptide flexibility, spatial orientation, and interaction with fungal surface components, potentially affecting adsorption, membrane association, or access to cellular targets [40].

The poor performance of peptide 2, FEFR, against all tested microorganisms further supports the importance of peptide length and residue distribution. Although this tetrapeptide contains phenylalanine, glutamate, and arginine, its very short sequence may limit the formation of an amphipathic structure and reduce its capacity to establish multiple interactions with microbial surfaces. In antimicrobial peptide design, short sequences can be active, but they generally require highly optimized arrangements of cationic and hydrophobic residues to compensate for their limited molecular size [41]. In the present study, peptide 2 showed MIC values above 3487.3 µmol L^−1^ for *S. aureus*, above 3487.3 µmol L^−1^ for *S. enterica*, and above 1918.1 µmol L^−1^ for *C. albicans*, indicating no relevant antimicrobial effect under the tested conditions.

The comparison between peptides 1, 3, 5, and 6 also indicates that subtle sequence modifications affected antimicrobial performance against *S. aureus*. Peptides 1 and 5 have the same molecular weight but differ in residue arrangement, while peptides 3 and 6 contain nine amino acids and additional lysine or arginine residues. Despite these differences, their MIC values against *S. aureus* were relatively similar, ranging from 1598.7 to 1782.0 µmol L^−1^. This suggests that, within this sequence family, increasing or rearranging lysine/arginine residues was not sufficient to substantially enhance antibacterial activity. The persistence of glutamate residues and the lack of a clearly optimized amphipathic motif may have limited membrane-disruptive efficiency [42].

From a food preservation perspective, the results indicate that the evaluated peptides are still at an early stage of screening. Their direct application as broad-spectrum preservatives is limited by the high MIC values against *S. aureus*, the absence of detectable activity against *S. enterica*, and the lack of bactericidal/fungicidal confirmation. However, the antifungal activity of peptide 4 against *C. albicans* is promising and may justify further investigation. This is particularly important since yeast contamination can compromise several food and beverage products, and antifungal preservatives with peptide-based structures may be attractive alternatives for clean-label or bioinspired preservation systems [9].

The balance between cationicity and hydrophobicity is a major determinant of antimicrobial peptide activity. Cationic residues promote initial attraction to microbial surfaces, while hydrophobic residues facilitate interaction with and insertion into lipid bilayers [37]. However, an appropriate balance is required, since insufficient cationicity can reduce peptide adsorption, whereas excessive hydrophobicity may increase nonspecific interactions and toxicity [39]. Structure-activity studies have shown that hydrophobicity, the distribution of positive charge, and amphipathic organization are central parameters controlling peptide interactions with microbial membranes. In this context, the modest antibacterial activity observed here may be associated with an incomplete optimization of these physicochemical properties [38].

In addition to amino acid composition, the antimicrobial performance of short peptides is widely reported to depend on a combination of physicochemical and structural parameters, including net charge, hydrophobicity, amphipathicity, residue distribution, and conformational behavior. Literature studies have shown that these factors must be balanced rather than maximized individually. For instance, Yang et al. [43] investigated α-helical peptide derivatives against *Candida albicans* and reported that increasing hydrophobicity by introducing aromatic residues enhanced antifungal efficacy, although excessive hydrophobicity also increased toxicity. Their results indicated that moderate hydrophobicity, rather than extreme charge or hydrophobicity alone, was more favourable for achieving selective antifungal activity.

Peptide secondary structure is another relevant parameter frequently associated with AMP performance. Circular dichroism (CD) spectroscopy has been widely employed to investigate whether antimicrobial peptides adopt ordered conformations under membrane-mimicking conditions. For example, Manzo et al. [44] reported that temporin L and aurein 2.5 adopted highly ordered α-helical conformations in membrane-mimicking media based on far-UV CD spectra. Likewise, Lee et al. [45] showed by CD analysis that both L-Ple and D-Ple displayed typical α-helical structures, although with different helicities associated with distinct biological effects. These observations indicate that differences in activity among peptides with related compositions may also reflect differences in conformational plasticity and membrane-induced folding, aspects that were not directly evaluated in the present study.

Proteolytic stability is also recognized as a critical determinant of peptide performance. Chen et al. [46] emphasized peptide stability as a major obstacle for AMP application and showed that partial D-amino acid substitution could preserve antimicrobial activity while improving the overall stability profile. In a more detailed degradation study, Lu et al. [47] demonstrated that D- and unnatural-amino-acid substitutions increased the stability of peptide 5 derivatives toward trypsin, plasma proteases, and microbial proteases, with cleavage occurring preferentially after L-Lys and L-Arg residues. Similarly, Zhao et al. [48] reported that replacement of trypsin-cleavage-site cationic residues by D-amino acids generated a derivative with maintained antibacterial activity and remarkably improved stability toward trypsin, serum, and plasma. Therefore, although the present study provides an initial screening based on sequence composition and biological activity, additional analyses of conformational behavior and proteolytic susceptibility would be valuable to better interpret the selective antifungal performance observed for peptide 4.

From an application-oriented perspective, several sequence and formulation strategies could be explored to improve the stability and performance of the most promising peptide identified in this study. Partial substitution of protease-sensitive L-amino acids by their D-enantiomers represents one particularly relevant approach, since previous studies have demonstrated that this strategy can substantially increase resistance to trypsin, serum, plasma, and microbial proteases while preserving antimicrobial [36]. For peptide 4, such substitutions could be investigated preferentially at residues susceptible to proteolytic cleavage, provided that the substitutions do not compromise the sequence features associated with its antifungal activity. Additional structural strategies, including peptide cyclization, may further restrict conformational freedom and reduce accessibility to proteolytic enzymes, potentially increasing peptide stability. At the formulation level, encapsulation or incorporation into micro- or nanostructured delivery systems could protect peptides from interactions with food components and environmental degradation while enabling controlled release at the site of application [40].

The sequence-derived physicochemical properties of the evaluated peptides are summarized in Table 2. In addition to peptide length and molecular weight, the comparison includes the number of basic and acidic residues, nominal net charge, phenylalanine content, and the grand average of hydropathy. These descriptors provide a more detailed basis for examining whether the observed antimicrobial profiles can be related to differences in charge balance, residue composition, and overall hydropathic character.

The evaluated sequences ranged from 4 to 15 amino acids, with molecular weights between 651.7 and 1960.2 g mol^−1^. Peptide 2, FEFR, was the shortest sequence and showed no detectable activity against any microorganism. This suggests that, despite containing phenylalanine and arginine residues, its reduced molecular size may limit the formation of a stable amphipathic arrangement and decrease the number of interactions required for efficient membrane destabilization. On the other hand, peptides 1, 3, 5, and 6, which contain 8 or 9 amino acids, showed inhibitory activity against *S. aureus*, although only at high concentrations. Peptide 4 also displayed the lowest phenylalanine proportion and one of the most negative GRAVY values, suggesting a more hydrophilic overall composition than the shorter sequences. Therefore, its selective activity may be more closely related to peptide length, residue arrangement, conformational adaptability, and the spatial distribution of interacting groups than to maximized cationicity or overall hydrophobicity.

A structure–activity relationship (SAR) comparison within the evaluated peptide set highlights three sequence features that appear particularly relevant to the observed antimicrobial profiles. First, peptide 2 (FEFR), the shortest sequence evaluated, showed no detectable activity against any of the tested microorganisms. Its tetrapeptide length likely limits the number of simultaneous interactions that can be established with microbial surfaces or molecular targets and may also restrict the development of a stable amphipathic arrangement [40]. Then, peptide 4 (FEFKFEFKGGGRGDS) was the only sequence showing relevant activity against *C. albicans*. In addition to being the longest peptide evaluated, it contains the GGGRGDS extension, in which the glycine-rich segment may increase backbone flexibility, and the RGDS motif introduces additional polar and charged residues. These features may promote a broader range of intermolecular contacts and facilitate adaptation to fungal surface components or protein-binding regions [9]. Furthermore, the recurrent presence of glutamate residues throughout the peptide set reduces the overall cationic character otherwise provided by lysine and arginine. This effect is particularly relevant for bacterial membrane interactions, since a lower positive charge density may weaken electrostatic attraction toward negatively charged cell-envelope components [49].

Among the evaluated sequences, peptide 4 displayed a distinct antifungal profile, being the only peptide capable of inhibiting *C. albicans*, with an MIC of 19.93 µmol L^−1^. Its activity cannot be attributed exclusively to the presence of cationic residues, since peptides containing a greater proportion of lysine and arginine did not produce comparable inhibition. Instead, the biological response appears to reflect the combined influence of peptide length, residue arrangement, and the spatial distribution of hydrophobic, polar, and charged groups. Peptide 4 is the longest sequence evaluated and contains the GGGRGDS segment, whose glycine-rich region may increase conformational flexibility, while the RGD motif introduces additional polar and charged residues. These characteristics may alter the exposure and organization of interacting groups and favor association with fungal envelope components or access to cellular targets. Nevertheless, these sequence-derived interpretations remain provisional because the peptides’ conformational behavior was not experimentally characterized [8].

The selective activity of peptide 4 against *C. albicans* is relevant, as antifungal antimicrobial peptides can act through multiple mechanisms, including interactions with the fungal cell wall, disruption of the plasma membrane, induction of intracellular stress, and interference with biofilm formation. Ma et al. [7] demonstrated, in their literature review on anti-Candida peptides, that antifungal activity depends heavily on sequence composition, charge, hydrophobicity, and structural organization. More recent studies by Schaefer et al. [50] with synthetic peptide mimics have also shown that positively charged peptide-like molecules can affect *C. albicans* by damaging cell wall-associated structures and inducing membrane-related effects. Therefore, the MIC obtained for peptide 4 suggests that this sequence may be a useful starting point for the rational development of antifungal peptide-based preservatives. However, the minimum fungicidal concentration of peptide 4 was not determined within the tested range [7]. Therefore, its activity should be interpreted as inhibitory rather than confirmed fungicidal. This distinction is important for food preservation, since fungistatic compounds may delay microbial proliferation but may not eliminate viable cells. For application in food systems, further assays should evaluate time-dependent killing, membrane permeability, peptide stability under food-processing conditions, and activity in model food matrices. Such studies are necessary because proteins, salts, lipids, and carbohydrates in foods may interact with peptides, reducing their antimicrobial activity [11].

### 3.2. Molecular Docking Analysis Against Candida albicans Target

**Figure 2:**
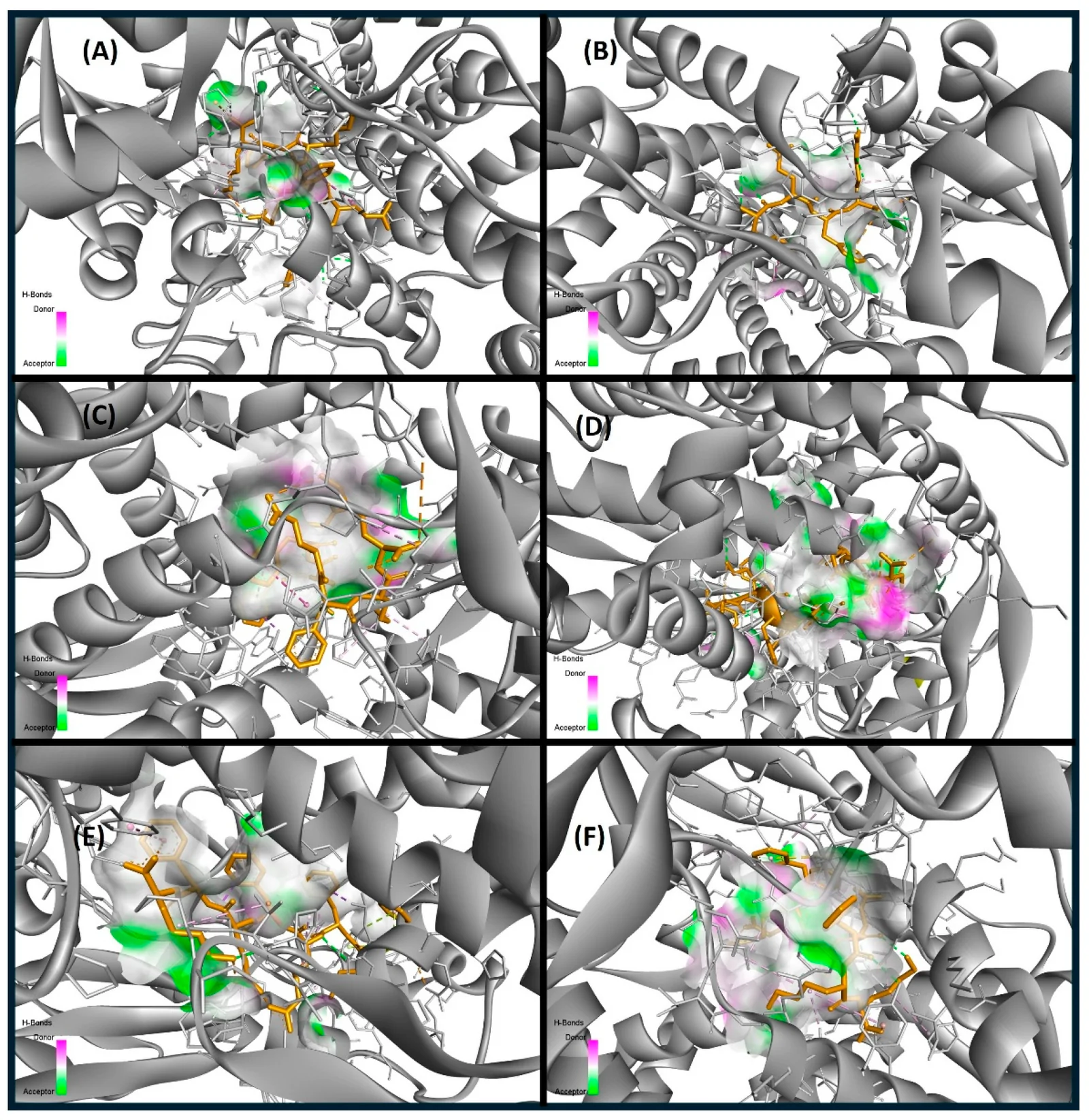
Docking poses of peptides 1–6 in complex with Candida albicans ERG11: peptide 1 (**A**), peptide 2 (**B**), peptide 3 (**C**), peptide 4 (**D**), peptide 5 (**E**), and peptide 6 (**F**). ERG11 is shown in grey representation, while the peptides and interacting residues are represented in orange.

Molecular docking was performed to compare the predicted interaction profiles of the six peptides with *C. albicans* ERG11. The analysis was restricted to a fungal target because peptide 4 was the only sequence showing relevant activity against *C. albicans*, whereas no inhibition was detected against *S. enterica* and only weak inhibition was observed against *S. aureus* at high peptide concentrations. ERG11 was selected because of its central role in ergosterol biosynthesis and its established relevance as a fungal pharmacological target. The amino acids that constitute the catalytic site of ERG11 include Lys143, Arg381, His468, Arg469, and Cys470. The docking scores are summarized in Table 3, while the highest-ranked poses and the main predicted peptide–receptor contacts are shown in Figure 2 and Table S1, respectively.

The docking results are summarized in Table 3. Since peptide 4 was the only sequence showing relevant in vitro activity against *C. albicans*, whereas no peptide inhibited *S. enterica* and only limited inhibition of *S. aureus* was observed at high concentrations, the docking analysis was restricted to a fungal target in order to explore the only biologically distinct activity pattern identified in the study. In this context, ERG11 was selected for its key role in ergosterol biosynthesis and its well-established status as a fungal pharmacological target.

Among the six peptides evaluated, peptide 4 yielded the lowest AutoDock Vina docking score (−8.4 kcal mol^−1^), followed by peptide 6 (−7.8 kcal mol^−1^). These values should be interpreted as comparative outputs of the Vina scoring function rather than experimentally determined binding free energy. Furthermore, the peptides evaluated ranged from 4 to 15 amino acids, so a direct comparison of absolute binding scores may be influenced by ligand size [51]. Larger peptides contain more atoms and potential interaction sites and may consequently establish a greater number of favorable contacts with the receptor. Therefore, the lower score obtained for peptide 4 cannot be attributed exclusively to stronger molecular recognition of ERG11 [52]. The docking scores obtained for peptides 4 and 6 were relatively close, differing by 0.6 kcal mol^−1^ (−8.4 and −7.8 kcal mol^−1^, respectively). This difference should be interpreted in the context of the distinct peptide sizes, since peptide 4 contains 15 residues whereas peptide 6 contains 9 residues, providing peptide 4 with a larger interaction surface and a greater number of potential receptor-contact points. Accordingly, the Vina scores were not interpreted as direct quantitative measures of binding affinity, but rather as comparative indicators of predicted peptide–ERG11 accommodation. Importantly, the interpretation of the docking results was also supported by the predicted interaction pattern, as peptide 4 established contacts with several functionally relevant ERG11 residues, including Lys143, Arg381, His468, Arg469, and Cys470. Thus, the computational analysis was considered on the basis of both docking score and receptor-contact profile, rather than on the numerical score alone.

Analysis of the predicted interaction network revealed that peptide 4 established contacts with several residues located within or adjacent to the functional region of ERG11. Electrostatic interactions were predicted with Lys143, His468, Cys470, and Arg469, while a hydrogen bond was identified with Arg381. The predicted contact with Cys470 is of particular interest since this conserved residue serves as the axial ligand of the heme iron in CYP51 enzymes. However, because the heme cofactor was not explicitly included in the AlphaFold-derived docking model, this interaction should be interpreted as a predicted contact with the protein environment of the heme-binding region rather than as evidence of direct interference with heme coordination. Peptide 4 also contacted His468 and Arg469, which are located in close proximity to Cys470, as well as Lys143 and Arg381, residues associated with the organization and stabilization of the heme-containing region. Additionally, these contacts indicate that peptide 4 was predicted to occupy a region of ERG11 encompassing residues of functional relevance, rather than interacting exclusively with a peripheral protein surface. Contacts with Cys470 were not exclusive to peptide 4. Peptides 5 and 6 also showed predicted hydrophobic contacts with this residue, at 4.2726 Å and 3.8434/5.2207 Å, respectively. Nevertheless, peptide 4 differed by establishing a broader interaction network involving multiple functionally relevant residues, including Lys143, Arg381, His468, Arg469, and Cys470. Thus, the distinctive profile of peptide 4 may be associated with the overall extent and distribution of predicted interactions within the ERG11 functional region, rather than with contact with Cys470 alone [53].

The superior docking performance of peptide 4 may be related to its higher molecular size and sequence composition. Peptide 4 is the longest sequence evaluated, containing 15 amino acids and the GGGRGDS segment, whereas the other peptides contain only 4 to 9 residues. The presence of glycine residues may increase conformational flexibility, allowing better accommodation of the peptide into the binding region of the fungal target. In addition, the RGD motif introduces polar and charged residues that may contribute to hydrogen bonding and electrostatic interactions [53]. The combination of phenylalanine residues, which can contribute to hydrophobic contacts, with lysine, arginine, aspartate, serine, and glycine residues may favor a more extensive interaction network than that observed for the shorter peptides. This interpretation is consistent with the general structure-activity principles described for antifungal antimicrobial peptides. Anti-Candida peptides frequently require an adequate balance between cationic charge, hydrophobicity, amphipathicity, and structural flexibility to interact with fungal cell envelopes and/or intracellular targets [54].

Perez-Rodriguez et al. [11] describe that their activity may involve membrane perturbation, interaction with cell wall components, oxidative stress induction, inhibition of morphogenesis, and interference with intracellular pathways. Thus, sequence composition and spatial distribution of charged and hydrophobic residues are decisive for antifungal performance. The MIC obtained for peptide 4, 19.93 µmol L^−1^, is also within a relevant range when compared with other antifungal peptide-based systems reported in the literature. For example, synthetic cationic peptides composed of 9–11 amino acids have been reported to inhibit *C. albicans* with MIC values in the range of 8–32 µg mL^−1^, corresponding approximately to 6–23 µM, depending on peptide structure and molecular weight. In this context, the activity of peptide 4 can be considered promising, particularly since it was not originally optimized as an antifungal sequence [50].

The lack of a direct correspondence between docking score and antifungal activity is particularly evident when comparing peptides 4 and 6. Although peptide 6 yielded the second-lowest AutoDock Vina score within the evaluated set, it did not inhibit *C. albicans* within the tested concentration range. This apparent discrepancy is expected because docking evaluates the predicted interaction of a ligand with an isolated receptor structure, whereas antimicrobial activity in vitro results from a sequence of additional physicochemical and biological events [55]. Before reaching an intracellular or membrane-associated molecular target, a peptide must remain sufficiently soluble and stable in the assay medium, interact productively with the fungal cell surface, cross or perturb the cell envelope, and retain an adequate concentration and conformation to access the target [11]. Peptide aggregation, proteolytic degradation, ionic interactions with medium components, limited membrane association, or inefficient cellular uptake may therefore reduce biological activity even when a favorable receptor-binding pose is predicted. Conversely, the detectable antifungal activity of peptide 4 likely reflects the combined contribution of these factors together with its predicted interaction profile with ERG11 [56].

Molecular docking was employed here as an exploratory computational approach to identify plausible peptide–target interactions and to provide a preliminary molecular rationale for the selective antifungal activity observed for peptide 4. This use of docking as a supportive, hypothesis-generating strategy is consistent with previous studies on antimicrobial peptides and antifungal compounds, although the proposed mode of action still requires validation through dedicated experimental studies. According to Flórez-Castillo et al. [57], molecular docking may be used as a first approximation to investigate possible AMP-target interactions and to support the interpretation of experimental antimicrobial results. Likewise, Agoni et al. [49] highlighted docking as a useful tool to predict peptide binding modes and potential interaction regions in bioactive peptide discovery. In the antifungal context, Sousa et al. [58] performed peptide docking against Candida albicans targets, including lanosterol 14-demethylase, to explore plausible molecular interactions associated with antifungal activity.

The combined experimental and computational results indicate that peptide 4 is the most promising candidate among the evaluated sequences for further investigation as an antifungal agent. Its selective activity against *C. albicans*, together with the most favorable docking score, suggests that this peptide may serve as a structural platform for rational optimization. Future modifications could aim to increase cationic density, improve amphipathic organization, enhance proteolytic resistance, and preserve the flexible region that may contribute to target accommodation.

## 4. Conclusions

Six short synthetic peptides were comparatively screened against *Staphylococcus aureus*, *Salmonella enterica*, and *Candida albicans*. The evaluated sequences showed a restricted and microorganism-dependent antimicrobial profile. No inhibition was observed against *S. enterica*, whereas peptides 1, 3, 5, and 6 inhibited *S. aureus* only at relatively high concentrations. Peptide 4 (FEFKFEFKGGGRGDS) was the only sequence showing relevant antifungal activity, inhibiting *C. albicans* at 19.93 µmol L^−1^. In the computational analysis, peptide 4 yielded the lowest AutoDock Vina docking score within the evaluated set (−8.4 kcal mol^−1^) and established predicted contacts with several functionally relevant ERG11 residues, including Lys143, Arg381, His468, Arg469, and Cys470. Thus, these findings identify peptide 4 as the most promising sequence within the evaluated peptide set for further antifungal investigation. The peptides were also evaluated in microbiological culture media rather than in food or food-mimicking matrices, and their structural behavior was not experimentally characterized by techniques such as circular dichroism. In addition, the docking results represent predicted peptide–ERG11 interaction patterns and should not be interpreted as direct evidence of ERG11 inhibition or as experimental binding affinities. Future research should proceed sequentially from peptide characterization and optimization toward application-oriented validation. Initial studies should confirm peptide identity, purity, structural behavior, proteolytic stability, and safety, while evaluating sequence modifications designed to improve activity and stability. Subsequent investigations should determine fungicidal activity, membrane permeabilization, time-dependent killing, and the possible involvement of ERG11 and ergosterol-associated pathways. Finally, the most promising optimized sequences should be evaluated in food-mimicking systems and model food matrices to assess stability, antimicrobial performance, formulation compatibility, and practical suitability for food-preservation applications.

## Figures and Tables

**Table 1:** Minimum inhibitory concentration values of the evaluated peptides against food-relevant microorganisms.

Peptide	Sequence	*Staphylococcus aureus* MIC (µmol L^−1^)	*Salmonella enterica* MIC (µmol L^−1^)	*Candida albicans* MIC (µmol L^−1^)
Peptide 1	FEFEFRFK	1782.0	>1782.0	>980.1
Peptide 2	FEFR	>3487.3	>3487.3	>1918.1
Peptide 3	FEFKFEFKK	1630.8	>1630.8	>897.0
Peptide 4	FEFKFEFKGGGRGDS	>1159.4	>1159.4	19.93
Peptide 5	FEFRFEFK	1782.0	>1782.0	>980.1
Peptide 6	FEFRFEFKK	1598.7	>1598.7	>879.3

**Table 2:** Theoretical properties of the evaluated short synthetic peptides.

Peptide	Number of Amino Acids	Molecular Weight (g mol^−1^)	Basic Residues	Acidic Residues	Phenylalanine Content (%)	GRAVY ^1^
Peptide 1	8	1275.4	2	2	50.0	−0.53
Peptide 2	4	651.7	1	1	50.0	−0.60
Peptide 3	9	1393.6	3	2	44.4	−0.83
Peptide 4	15	1960.2	3	3	26.7	−0.93
Peptide 5	8	1275.4	2	2	50.0	−0.53
Peptide 6	9	1421.6	3	2	44.4	−0.90

^1^ GRAVY, grand average of hydropathy, was calculated using the Kyte–Doolittle hydropathy scale. More negative values indicate an overall more hydrophilic sequence.

**Table 3:** AutoDock Vina docking scores obtained for the evaluated peptides with *Candida albicans* ERG11.

Peptide	Sequence	Docking Score
Peptide 4	FEFKFEFKGGGRGDS	−8.4 kcal mol^−1^
Peptide 6	FEFRFEFKK	−7.8 kcal mol^−1^
Peptide 5	FEFRFEFK	−6.9 kcal mol^−1^
Peptide 3	FEFKFEFKK	−6.3 kcal mol^−1^
Peptide 2	FEFR	−5.8 kcal mol^−1^
Peptide 1	FEFEFRFK	−5.4 kcal mol^−1^

## Data Availability

The original contributions presented in this study are included in the article/Supplementary Material. Further inquiries can be directed to the corresponding author.

## Supplementary Materials

The following supporting information can be downloaded at https://www.mdpi.com/article/10.3390/microorganisms14092088/s1. Table S1: Docking affinity energy and interacting atoms predicted by AutoDock Vinna between ERG11 from *Candida albicans* and peptides. Figure S1: Determination of the minimum non-toxic concentration (MNTC). Representative experimental plate used for MNTC determination. Figure S2: Representative RP-HPLC chromatograms of the peptide sequences investigated in this study. Peptide samples (1 mg mL^−1^) were prepared in a 1:1 (*v*/*v*) mixture of water and acetonitrile containing 1% trifluoroacetic acid (TFA). Chromatographic separation was performed on a Phenomenex Jupiter Proteo 90 Å column (4 µm, 250 × 4.6 mm) with UV detection at 220 nm. Elution was carried out at a flow rate of 1.0 mL min^−1^ using a linear gradient from 90% solvent A/10% solvent B to 30% solvent A/70% solvent B over 45 min, where solvent A consisted of 0.05% TFA in H_2_O and solvent B consisted of 0.05% TFA in CH_3_CN.
