# Supplementary material for: In Vitro Screening of Short Synthetic Antimicrobial Peptides Toward Food-Relevant Microorganisms: Antifungal Activity and Molecular Docking Analysis
Source: Microorganisms. 2026 Sep 18;14(9):2088. doi: 10.3390/microorganisms14092088 (PMC13609430; doi:10.3390/microorganisms14092088)
Supplement: Supplementary file 1 [file microorganisms-14-02088-s001.zip › microorganisms-4521406-supplementary.pdf]

Supporting Information

In vitro screening of short synthetic antimicrobial peptides toward food-relevant microorganisms: antifungal activity and molecular docking analysis

Marselle Marmo do Nascimento Silva[1], Aline F. Miller Gavin Humphreys[2], Maria Alice Zarur Coelho[1], Filipe Smith Buarque[3,*]

[1] Biochemical Engineering Department, School of Chemistry, Federal University of Rio de Janeiro, Brazil. marselle@eq.ufrj.br; alice@eq.ufrj.br

[2] Faculty of Science and Engineering, University of Manchester, United Kingdom. aline.miller@manchester.ac.uk.

[3] CICECO-Aveiro Institute of Materials, Department of Chemistry, University of Aveiro, Portugal.

* Correspondence: filipesbuarque@ua.pt

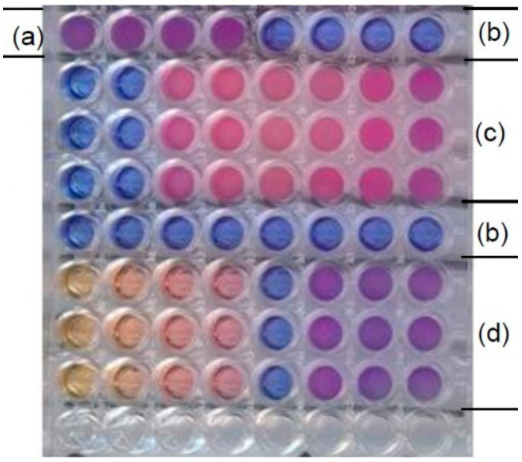

Figure S1. Determination of the minimum non-toxic concentration (MNTC). Representative experimental plate used for MNTC determination.

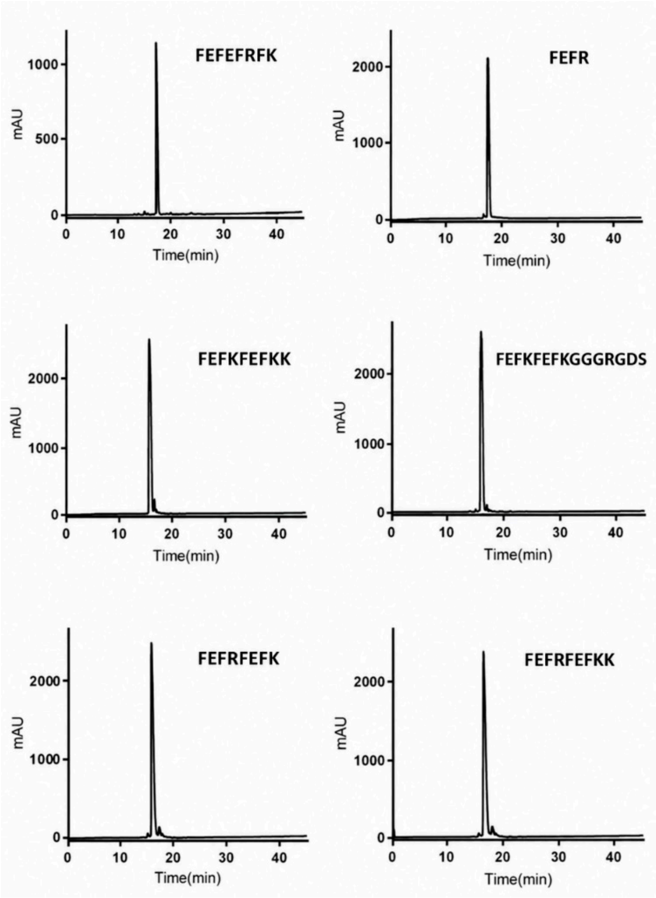

Figure S2. Representative RP-HPLC chromatograms of the peptide sequences investigated in this study. Peptide samples (1 mg mL^{-1}) were prepared in a 1:1 (v/v) mixture of water and acetonitrile containing 1% trifluoroacetic acid (TFA). Chromatographic separation was performed on a Phenomenex Jupiter Proteo 90 Å column (4 µm, 250 × 4.6 mm) with UV detection at 220 nm. Elution was carried out at a flow rate of 1.0 mL min^{-1} using a linear gradient from 90% solvent A/10% solvent B to 30% solvent A/70% solvent B over 45 min, where solvent A consisted of 0.05% TFA in H_2O and solvent B consisted of 0.05% TFA in CH_3CN.

Table S1: Docking affinity energy and interacting atoms predicted by AutoDock Vinna between ERG11 from *Candida albicans* and peptides.

Ligands	Affinity (kcal mol^{-1})	Amino acids (AA)	Distance (Å)	Binding types
Peptide 1	−5.4	LYS8	3.6064	Electrostatic
		LEU376	2.8932	Hydrogen Bond
		SER378	2.9164	Hydrogen Bond
		GLY464	2.5509	Hydrogen Bond
		PHE1	2.7903	Hydrogen Bond
		PHE1	2.2364	Hydrogen Bond
		PHE5	3.1719	Hydrogen Bond
		ARG6	3.1055	Hydrogen Bond
		ARG6	2.8253	Hydrogen Bond
		PHE7	3.2811	Hydrogen Bond
		PRO375	3.5988	Hydrogen Bond
		HIS377	3.3558	Hydrogen Bond
		LYS8	3.9318	Electrostatic
		HIS373	4.2999	Hydrophobic
		PRO406	5.4836	Hydrophobic
		ARG6	3.4851	Hydrophobic
		LYS8	4.0724	Hydrophobic
		LYS8	5.0749	Hydrophobic
		PHE1	4.8226	Hydrophobic
		PHE1	4.6971	Hydrophobic
		PHE3	5.1453	Hydrophobic
		PHE5	5.2263	Hydrophobic
		PHE5	3.6294	Hydrophobic
Peptide 2	−5.8	ARG381	5.2484	Electrostatic
		TYR118	3.1614	Hydrogen Bond
		THR315	3.3558	Hydrogen Bond
		PHE1	3.1823	Hydrogen Bond
		ARG4	2.7082	Hydrogen Bond
		ARG4	2.4480	Hydrogen Bond
		MET374	3.3515	Hydrogen Bond
		ARG4	4.5778	Electrostatic
		HIS310	4.5950	Hydrophobic
		ARG4	5.0243	Hydrophobic
		ARG4	3.8412	Hydrophobic
Peptide 3	−6.3	LYS143	5.3372	Electrostatic
		ARG469	3.6954	Electrostatic
		PHE1	4.9169	Electrostatic
		LYS9	4.1145	Electrostatic
		PHE5	3.3358	Hydrogen Bond
		LYS8	2.3204	Hydrogen Bond

		LYS4	3.5791	Hydrogen Bond
		LEU370	3.6168	Hydrophobic
		PHE3	5.7043	Hydrophobic
		PHE463	4.8852	Hydrophobic
		PRO406	4.1927	Hydrophobic
		PRO462	4.8177	Hydrophobic
		ARG469	5.1711	Hydrophobic
		LYS4	4.2699	Hydrophobic
		LYS4	4.0091	Hydrophobic
		PHE1	5.0431	Hydrophobic
		PHE3	4.2540	Hydrophobic
Peptide 4	−8.4	LYS147	3.677	Hydrogen Bond
		LYS143	3.677	Electrostatic
		HIS468	3.127	Electrostatic
		CYS470	3.682	Electrostatic
		ARG469	5.085	Electrostatic
		ARG469	5.216	Electrostatic
		LYS4	4.832	Electrostatic
		LYS8	3.753	Electrostatic
		ARG381	3.194	Hydrogen Bond
		LYS4	3.085	Hydrogen Bond
		LYS4	2.995	Hydrogen Bond
		PHE5	3.168	Hydrogen Bond
		PHE5	3.223	Hydrogen Bond
		GLU6	3.005	Hydrogen Bond
		PHE7	3.161	Hydrogen Bond
		LYS8	3.073	Hydrogen Bond
		LYS8	3.381	Hydrogen Bond
		GLY10	3.104	Hydrogen Bond
		ARG12	3.152	Hydrogen Bond
		SER15	2.550	Hydrogen Bond
		SER15	3.164	Hydrogen Bond
		PRO512	3.295	Hydrogen Bond
		PHE1	3.982	Electrostatic
		PHE1	3.272	Hydrophobic
		TYR505	5.199	Hydrophobic
		GLY13	3.350	Hydrophobic
		ASP14	3.350	Hydrophobic
		PHE1	4.241	Hydrophobic
		PHE3	5.347	Hydrophobic
		PHE5	4.570	Hydrophobic
Peptide 5	−6.9	PHE1	3.6310	Hydrogen Bond
		LYS8	2.6177	Electrostatic
		ARG4	2.9651	Hydrogen Bond

		LYS8	2.3740	Hydrogen Bond
		HIS377	2.9651	Hydrogen Bond
		HIS410	3.3708	Hydrogen Bond
		LYS8	4.8986	Electrostatic
		LEU370	3.9226	Hydrophobic
		LEU376	3.7233	Hydrophobic
		CYS470	4.2726	Hydrophobic
		LYS367	2.7901	Hydrophobic
		TYR460	2.9075	Hydrophobic
		PHE1	2.9895	Hydrophobic
		PHE228	5.8792	Hydrophobic
		TRP427	5.8233	Hydrophobic
		VAL509	5.3754	Hydrophobic
		ARG4	5.4691	Hydrophobic
		ARG4	5.1371	Hydrophobic
		LYS8	5.0396	Hydrophobic
		HIS410	4.2401	Hydrophobic
		PHE1	5.0922	Hydrophobic
		PHE3	3.9558	Hydrophobic
Peptide 6	−7.8	ARG4	3.3678	Hydrogen Bond
		PHE1	4.0150	Electrostatic
		TYR477	3.0316	Hydrogen Bond
		PHE5	2.9486	Hydrogen Bond
		PHE7	3.1403	Hydrogen Bond
		LYS9	2.5209	Hydrogen Bond
		GLU6	3.6319	Hydrogen Bond
		LYS9	4.5308	Electrostatic
		THR315	3.7994	Hydrogen Bond
		LEU376	3.9591	Hydrophobic
		MET374	5.3341	Hydrophobic
		CYS470	3.8434	Hydrophobic
		CYS470	5.2207	Hydrophobic
		ALA476	3.8162	Hydrophobic
		LYS9	4.8937	Hydrophobic
		LYS9	3.4657	Hydrophobic
		TYR477	5.4792	Hydrophobic
		PHE1	4.8979	Hydrophobic
		PHE3	5.0166	Hydrophobic
		PHE3	5.4319	Hydrophobic
		PHE5	4.9303	Hydrophobic
